# When noise makes music: HIV reactivation with transcriptional noise enhancers

**DOI:** 10.1186/s13073-014-0055-9

**Published:** 2014-07-29

**Authors:** Xu Tan, Stephen J Elledge

**Affiliations:** School of Medicine, Tsinghua-Peking Center for Life Sciences, Tsinghua University, Beijing, 100084 China; Department of Genetics, Harvard Medical School, Howard Hughes Medical Institute, Division of Genetics, Brigham and Women’s Hospital, Boston, MA 02115 USA

## Abstract

Reactivating latent HIV is key to depleting the virus reservoir in AIDS patients. A recent paper has described the rationale for and discovery of a new class of drugs - transcriptional noise enhancers - that can synergize with conventional transcription activators to more effectively reactivate latently infected T cells. As well as describing a promising new strategy in the bid to find a cure for AIDS, this study more broadly highlights the utility of exploring drug combinations in treatment of human disease.

It has been over 30 years since HIV was discovered as the cause of AIDS, which has taken over 30 million lives, but we have yet to find a vaccine or a cure for this disease. Developing a cure for AIDS has been hampered by the difficulty of eradicating quiescent viruses that are integrated into the genomes of long-lived CD4^+^ memory T cells but are inactive for gene expression and replication [1]. These so-called latent viruses can persist over long periods of time and can be reactivated under certain circumstances to initiate new rounds of replication [2]. HIV-infected individuals therefore need life-long treatment to suppress viral replication.

A leading strategy that is being followed to develop a cure for AIDS is to reactivate the latent viruses so that the reservoir of latently infected T cells will be killed by either the replicating viruses or the immune system (the ‘shock and kill’ strategy) [3]. In combination with anti-retroviral therapy (ART), the reactivation strategy might enable the eradication of the virus in patients. However, currently available small molecules capable of reactivating HIV have been shown to be insufficient to fully activate the latently infected T cells in cell-based assays and clinical trials [4,5]. More potent small molecules and their combinations are clearly needed to realize the potential of the shock and kill strategy.

Dar *et al.* now report in *Science* [6] that a surprising class of small molecules might help the currently available HIV-reactivating drugs to further enhance reactivation efficiency. Single-cell gene expression analysis has demonstrated a non-uniform distribution of gene expression in otherwise identical cells. The variance of gene expression from the mean is referred to as noise; the greater the variation, the greater the noise. The class of molecules identified by Dar *et al.* [6] increases the variation of transcriptional activity (transcriptional noise) of a reporter under the control of the HIV long terminal repeat (LTR) promoter. In contrast to conventional transcriptional reactivators, these noise enhancers do not alter the mean transcriptional activity.

Based on computational simulations, Dar *et al.* [6] reasoned that increasing the noise level of gene expression might have synergistic effects with conventional transcriptional reactivators to enhance HIV reactivation from latency. Following on from this hypothesis, the authors [6] screened for enhancers of noise from a library of 1,600 bioactive small molecules. The screen used a Jurkat cell line (immortalized human T lymphocytes) with an integrated copy of a short-lived green fluorescent protein gene (*d2GFP*) driven by the LTR promoter of HIV, as the short half-life of the fluorescent protein faithfully reports on transcriptional efficiency [7].

The authors identified 110 compounds that showed noise enhancing activity but not direct transcription up-regulation activity. They further tested the activity of these molecules in a dual reporter system expressing the short-lived d2GFP and a stable mCherry fluorescent protein. Based on their computational model, the noise level of the abundance of a stable protein is only minimally affected by transcriptional noise. With this in mind, the authors [6] eliminated those compounds that affect the noise of both d2GFP and mCherry expression, leaving 85 compounds for further analysis. By monitoring single cells with a broad spectrum of integration sites, they confirmed that these compounds increase transcriptional noise regardless of integration site. This is important because the ability to affect the noise at all expression levels means that potentially latent HIV integrants in many genomic locations and with different basal levels of expression may all be responsive to these compounds.

Remarkably, the authors [6] found that the majority of the 85 compounds demonstrated synergistic reactivation activities with molecules that have conventionally been used as transcriptional activators of latent HIV (tumor necrosis factor (TNF), prostratin or phorbol esters such as PMA) in three latently infected cell lines and in primary T cells. Just as the authors’ theory predicted [6], the degree of synergy for a given compound shows a strong correlation with the magnitude of noise enhancement, suggesting that the synergy indeed stems from the noise enhancing activity. In the other direction, a noise suppressor was also tested in combination with TNF and shown to antagonize the reactivation activity of TNF.

Notably, the cytotoxicity of the combinations of noise enhancers and transcriptional activators is significantly lower than that of a leading combination of transcriptional activators (SAHA plus prostratin) with a similar degree of reactivation efficiency. This suggests an improved therapeutic window of the noise enhancer/transcription activator combinations. HIV reactivation might be particularly well-suited as a target for this strategy because the goal is the activation of a forward-feeding transcriptional loop that involves the viral Tat protein and the TAR RNA. Increasing the noise increases the frequency with which a cell might surpass the threshold needed to flip the Tat-TAR circuit on, which then maintains itself.

What is the mechanism of noise enhancement? The authors report that many known chromosome remodelers, such as histone deacetylase inhibitors, DNA methylation inhibitors and bromodomain inhibitors, can increase transcriptional noise. This property could be explained by their ability to make the chromosome contexts more accessible to the transcriptional machinery. The top candidates from the screen, however, have diverse known functions that include microtubule inhibitors, an anti-inflammatory agent, an estrogen receptor ligand and a DNA/RNA synthesis inhibitor. It is not obvious how such an array of compounds can each work specifically to increase the transcriptional noise of HIV. Plausible explanations include off-target effects of these compounds or that there are many regulatory pathways that can modulate HIV transcription. Three cell cycle inhibitors, including two microtubule depolymerization inhibitors and a DNA/RNA synthesis inhibitor, are among the top 10 hits, which seems to suggest that cell cycle inhibition might have a role in the noise enhancement. Further studies are needed to elucidate the mechanisms of action of these noise enhancing compounds, which will shed light on how transcription noise is controlled in general.

In this study, Dar *et al.* [6] developed a novel approach to screen for synergistic HIV reactivating drug combinations. This approach - noise enhancement - may have broader implications for drug discovery targeting other diseases. This work also highlights the power and necessity of systematically searching for drug combinations rather than combining drugs with the desired activity individually. This is clearly demonstrated by the fact that most of the noise enhancers have no effect on reactivation individually so they would have been missed by conventional single drug screenings. Complete HIV reactivation is a tall order unlikely to be achieved by a single agent. Neither the current leading cocktails nor the noise enhancer plus transcriptional activator combinations developed in this study can fully reactivate the latently infected cells *in vitro*. It might be even more difficult to reactivate latent virus *in vivo* given that certain tissues harboring latent virus, such as the central nervous system and gut-associated lymphoid tissue, are difficult to penetrate with drugs [4]. Drug combinations with new activities and innovative drug delivery methods will likely be needed to accomplish this task.

Importantly, mere screening for reactivation might be insufficient as the ultimate goal is to kill the reactivated cells. An ideal drug cocktail would be able to reactivate and promote the death of the reactivated cells. Recent new methods of high throughput screening [8] and immunotherapy [9,10] might be helpful to achieve this lofty goal. This eye-opening approach developed by Dar *et al.* [6] reminds us to keep an open mind to all possibilities in the search to end the AIDS pandemic.
